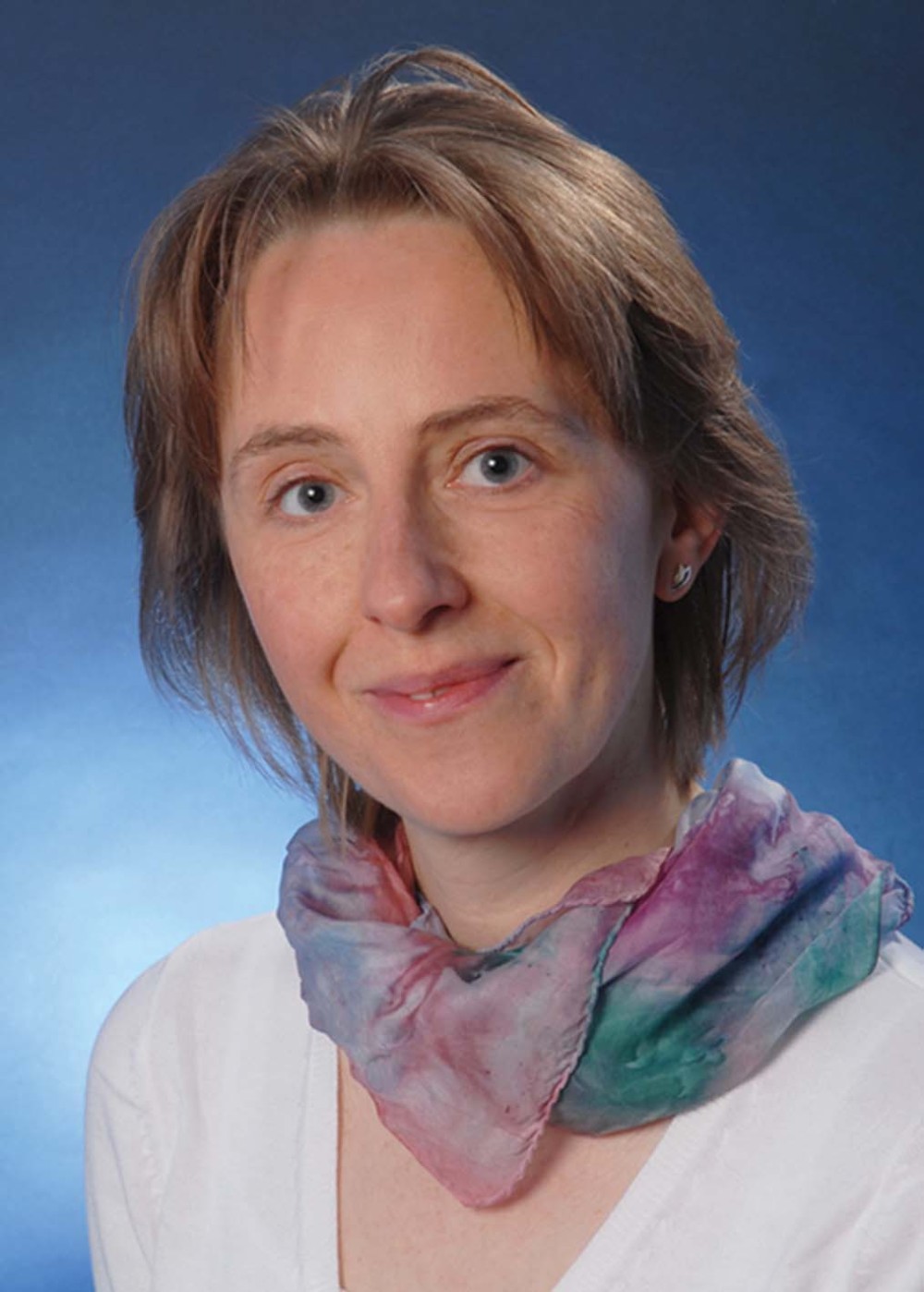# Gene therapy enters the pharma market: The short story of a long journey

**DOI:** 10.1002/emmm.201202291

**Published:** 2013-01-03

**Authors:** Hildegard Büning

**Affiliations:** Department I of Internal Medicine, Center for Molecular Medicine Cologne (CMMC), University of Cologne, CMMC Research BuildingCologne, Germany

Approximately 20 years ago, gene therapy was first introduced when Michael Blaese and colleagues applied *ex vivo* modified autologous T cells to children suffering from adenosine deaminase deficiency (ADA-SCID), a Mendelian genetic error that causes severe combined immunodeficiency syndrome (Blaese et al, 1995). The investigators used retroviral vectors; the most advanced gene delivery system at that time. They transferred a normal copy of adenosine deaminase that, according to the intrinsic features of retroviruses, stably integrated into the cellular genome. While this study demonstrated a proof of concept, years of tremendous efforts both in basic and translational research followed before the first clear evidence for a cure of SCID patients through gene therapy could be reported (Cavazzana-Calvo et al, 2012).

»In November 2012, the European Commission has granted marketing authorization for the first gene-therapy medicine…«

Now gene therapy in Europe has taken the next step. In November 2012, the European Commission has granted marketing authorization for the first gene-therapy medicine, Glybera® (alipogene tiparvovec), in all 27 European Union member states (http://www.uniqure.com). Glybera has been developed by uniQure (former Amsterdam Molecular Therapeutics) for the treatment of patients with familial lipoprotein lipase deficiency (LPLD, synonym: type I hyperlipidaemia).

LPLD is an ultra-rare disease with a prevalence of 1–2 in 10^6^ individuals in the general populations (Brunzell, 1999). It is an autosomal recessive disease found in all ethnic groups and it is caused by mutations in the *LPL* gene (chromosome 8p21.3). This gene encodes lipoprotein lipase (LPL), a key enzyme in the catabolism of triglyceride (TG)-rich lipoproteins that is secreted from adipocytes and muscle cells. In LPLD, TG-rich lipoproteins accumulate in the plasma leading to chylomicronemia and severe hypertriglyceridemia (TG concentration > 20 mmol/L). The latter causes episodes of abdominal pain, recurrent acute and potentially life-threatening pancreatitis, eruptive cutaneous xanthomata and hepatosplenomegaly (Brunzell, 1999). In the absence of any causal treatment, LPLD patients are advised to stay on a strict diet that reduces dietary fat to <20% of total caloric intake (http://www.ema.europe.eu). Although this diet is difficult to adhere to, it allows the majority of LPLD patients to stay free of symptoms (Brunzell, 1999).

Glybera is an adeno-associated viral (AAV) vector of serotype 1 encoding a naturally occurring gain-of-function variant of LPL, LPL^S447X^, *i.e.* found in 20% of Caucasians and, *i.e.* associated with lower plasma TG levels and a lower incidence of cardiovascular diseases (Gaudet et al, 2012a). Glybera is applied as local *in vivo* gene therapy. Specifically, it is injected under spinal anaesthesia at multiple sites in the muscles of the lower limbs. Choosing muscles as target site for the application has the advantage of good accessibility and of being the natural site of LPL expression. AAV1 vectors are characterized by a strong tropism for muscle tissue (Mingozzi & High, 2011), where in the case of Glybera, transgene expression controlled by a constitutively active promoter (Cytomegalovirus immediate early promoter) is initiated (Gaudet et al, 2012b).

AAV vectors are currently considered to be the best-suited vector system for long-term transgene expression in post-mitotic tissue (Buning et al, 2008; Mingozzi & High, 2011). Thus, besides gene transfer into skeletal muscle as with Glybera, AAV vectors are tested in liver-, lung-, heart-, eye- and central nervous system-directed human clinical trials (Jessup et al, 2011; Mingozzi & High, 2011; Nathwani et al, 2011). They are of low immunogenicity and persist as episomal concatemers, two features with a clear impact on vector safety (Buning et al, 2008; Mingozzi & High, 2011). In line with this, clinical trials have demonstrated an excellent safety profile.

Developing a novel treatment for an ultra-orphan disease such as LPLD is a difficult task. Following proof-of-concept in animal models, a clinical development program was launched that consisted of two observational studies, three open-label interventional studies and a long-term follow-up review analysis. While the observational studies aimed to determine baseline manifestation of the disease and impact of a controlled low-fat diet on chylomicronemia and plasma TG levels, a total of 27 LPLD patients received AAV-LPL^S447X^ within the three open-label interventional trials (Gaudet et al, 2012b).

The first interventional trial, CT-AMT-010-01, involved eight patients with homozygous mutations in the *LPL* gene that received either 1 × 10^11^ or 3 × 10^11^ vector genome copies (gc) per kg (Stroes et al, 2008). A reduction of plasma TG level (≤10 mmol/L or ≥40% compared to baseline) in patients on diet was defined as a primary endpoint. At 12 weeks, all eight patients showed a decrease in the TG levels, but only in 50% of the patients, the majority of which had received the higher vector dose, the primary endpoint was reached. This effect was transient, as TG levels had returned to baseline after 18–31 months. The second study, CT-AMT-011-01, included 14 patients, all presenting either a homozygous P207L or a heterogeneous P207L/G188L or P207L/D9N mutation, who either received 3 × 10^11^ or 1 × 10^12^ gc/kg (Gaudet et al, 2012a). Assuming that return to TG baseline levels observed in the first trial was related to a vector capsid-specific T-cell response that was detected in four of the eight patients (Mingozzi et al, 2009), an immunosuppressive regime (cyclosporine A and mycophenolate mofetil) was introduced (Gaudet et al, 2012b). Again, ≥40% reduction of TG levels was defined as a primary endpoint, which was reached by 50% of the patients. By week 16–26, however, as in the first trial, TG levels had returned to baseline levels in all patients, clearly indicating that the therapeutic effect of alipogene tiparvovec on TG levels was not persistent. When expanding the analysis to additional markers, signs for efficacy beyond week 12 could be reported. These included: (i) sustained modification of TG-rich lipoprotein characteristics, which were independent of the effect of alipogene tiparvovec on total TG levels; (ii) persistence of transgene expression; (iii) improvement of clinical symptoms as reported by the patients and (iv) reduced incidence and/or intensity of pancreatitis (Gaudet et al, 2012a, b). As a result of these findings, the third trial, CT-AMT-011-02, conducted with five LPLD patients with a P207L mutation in the *LPL* gene, focused on the postprandial chylomicron metabolism. It revealed that in all patients throughout the postprandial period the TG content of the chylomicron fraction and the chylomicron-triglyceride to total plasma TG ratio were reduced (Carpentier et al, 2012). Additionally, incidence and severity of pancreatitis and abdominal pain in LPLD patients before and after alipogene tiparvovec treatment were assessed in a long-term follow up study (CT-AMT-011-03), which demonstrated clinical benefit (Gaudet et al, 2012b).

Based on the data obtained in the clinical development program, the European Medicines Agency (EMA) finally recommended marketing authorization for alipogene tiparvovec (now Glybera®), followed by its approval by the European Commission. Although it was for a long time speculated that alipogene tiparvovec could become the first gene therapy medicine, its marketing authorization process was a long and winding road (http://www.ema.europa.eu/). It started with the initial submission in December 2009 that was turned down by the Committee for Medicinal Products for Human Use (CHMP) and the Committee for Advanced Therapy (CAT) in June 2011. Also during re-examination later that year, one of the two committees maintained its negative position. Finally, in January 2012, when the European Commission asked for re-evaluation of the application of Glybera for a more restricted use, namely for LPLD patients with severe and multiple attacks of pancreatitis despite dietary fat restrictions, both CHMP and CAT gave their consent. This complex process clearly illustrates the challenges of dealing with an ultra-orphan disease and thus very small patient numbers. Furthermore, the complexity of the dataset and the definition of the endpoints were likely important issues.

Although EU approval for the use of Glybera is now narrower than originally planned by uniQure and the regulatory authorities obliged the company to monitor and report on the patients' outcome (Watts, 2012), it is a huge step forward for the field and a clear signal to the public.

»… it can clearly be expected that more gene therapy-based therapeutics will make their way into clinical applications in the near future«

The perspectives offered by Gene Therapy for improving the treatment options for patients suffering from inherited or acquired diseases are accepted in the scientific community and beyond. A good measure for the latter is the increasing interests of companies in Gene Therapy. Nobody will deny that there is still a lot of work to do, however, it is also evident that reports on clinical benefit achieved by Gene Therapy have significantly increased in number during recent years (Cavazzana-Calvo et al, 2012; Kay, 2011; Nathwani et al, 2011). Thus, marketing authorization of a gene therapy medicine in the EU was expected by many to come for some time. Glybera is now the first gene therapy medicine on the EU market, but it can clearly be expected that more gene therapy-based therapeutics will make their way into clinical applications in the near future.

**Hildegard Büning**